# Sex differences in auditory processing vary across estrous cycle

**DOI:** 10.1038/s41598-021-02272-5

**Published:** 2021-11-24

**Authors:** Jennifer Krizman, Elena K. Rotondo, Trent Nicol, Nina Kraus, Kasia M. Bieszczad

**Affiliations:** 1 Auditory Neuroscience Laboratory, Northwestern University, Evanston, IL 60208 USA; 2 Department of Communication Sciences and Disorders, Northwestern University, Evanston, IL 60208 USA; 3 Department of Neurobiology, Northwestern University, Evanston, IL 60208 USA; 4 Department of Otolaryngology, Northwestern University, Chicago, IL 60611 USA; 5 Department of Psychology—Behavioral and Systems Neuroscience, Rutgers, The State University of New Jersey, Piscataway, NJ 08854 USA

**Keywords:** Auditory system, Sensory processing

## Abstract

In humans, females process a sound’s harmonics more robustly than males. As estrogen regulates auditory plasticity in a sex-specific manner in seasonally breeding animals, estrogen signaling is one hypothesized mechanism for this difference in humans. To investigate whether sex differences in harmonic encoding vary similarly across the reproductive cycle of mammals, we recorded frequency-following responses (FFRs) to a complex sound in male and female rats. Female FFRs were collected during both low and high levels of circulating estrogen during the estrous cycle. Overall, female rodents had larger harmonic encoding than male rodents, and greater harmonic strength was seen during periods of greater estrogen production in the females. These results argue that hormonal differences, specifically estrogen, underlie sex differences in harmonic encoding in rodents and suggest that a similar mechanism may underlie differences seen in humans.

## Introduction

In humans, auditory processing differs physiologically between males and females. These differences chiefly manifest in the timing and harmonic encoding of the response to sound, with females having earlier and larger responses than males^1,2^. Some timing differences are present at birth and are thought to result from differences in cochlear or auditory tract length^3,4^, while others do not emerge until adolescence, suggesting a hormonal source for these differences^1,5^. Animal work supports a role of hormones, specifically estrogen, in these sex differences^6^ as ovariectomized female rats have later response timing to sound than intact females, but the difference disappears with estrogen administration^7^.

Differences in harmonic encoding between males and females similarly do not arise until adolescence^1^. The change in harmonic encoding is greater than would be expected by differences in tract length (as inferred by differences in head size) between the sexes^1,8^, implicating hormonal differences emerging during adolescence. In support of a hormonal influence on harmonic encoding, estradiol treatment of non-breeding female midshipman fish amplifies their peripheral sensitivity to the harmonics of the male fish’s calls, akin to the enhanced harmonic response seen in breeding midshipman females^9^. These systematic fluctuations with piscine estrogen level provide evidence of a link between hormone levels and harmonic encoding in seasonally-breeding animals. However, it is unknown whether hormones similarly regulate harmonic processing in the central auditory system of mammals, or if subtle, natural, hormone variation across the female cycle can elicit these changes in harmonic encoding. The goal of this study was to determine whether male and female rodents show differences in harmonic encoding, similar to that seen in humans, and whether the magnitude of these differences vary with changes in hormone levels across the female estrous cycle.

## Results

To investigate whether differences in harmonic encoding vary between sexes and across the reproductive cycle of rodents, we recorded frequency-following responses (FFRs) to a 40-ms synthesized complex sound “da” in anesthetized male (n = 8) and female (n = 8) adult rats. Female FFRs were collected during both low and high levels of circulating estrogen during the estrous cycle. A subsequent analysis on FFRs from 5 additional male rates was also performed to verify that differences seen in females during the two recordings could not be attributed to test–retest differences.

**Figure 1:**
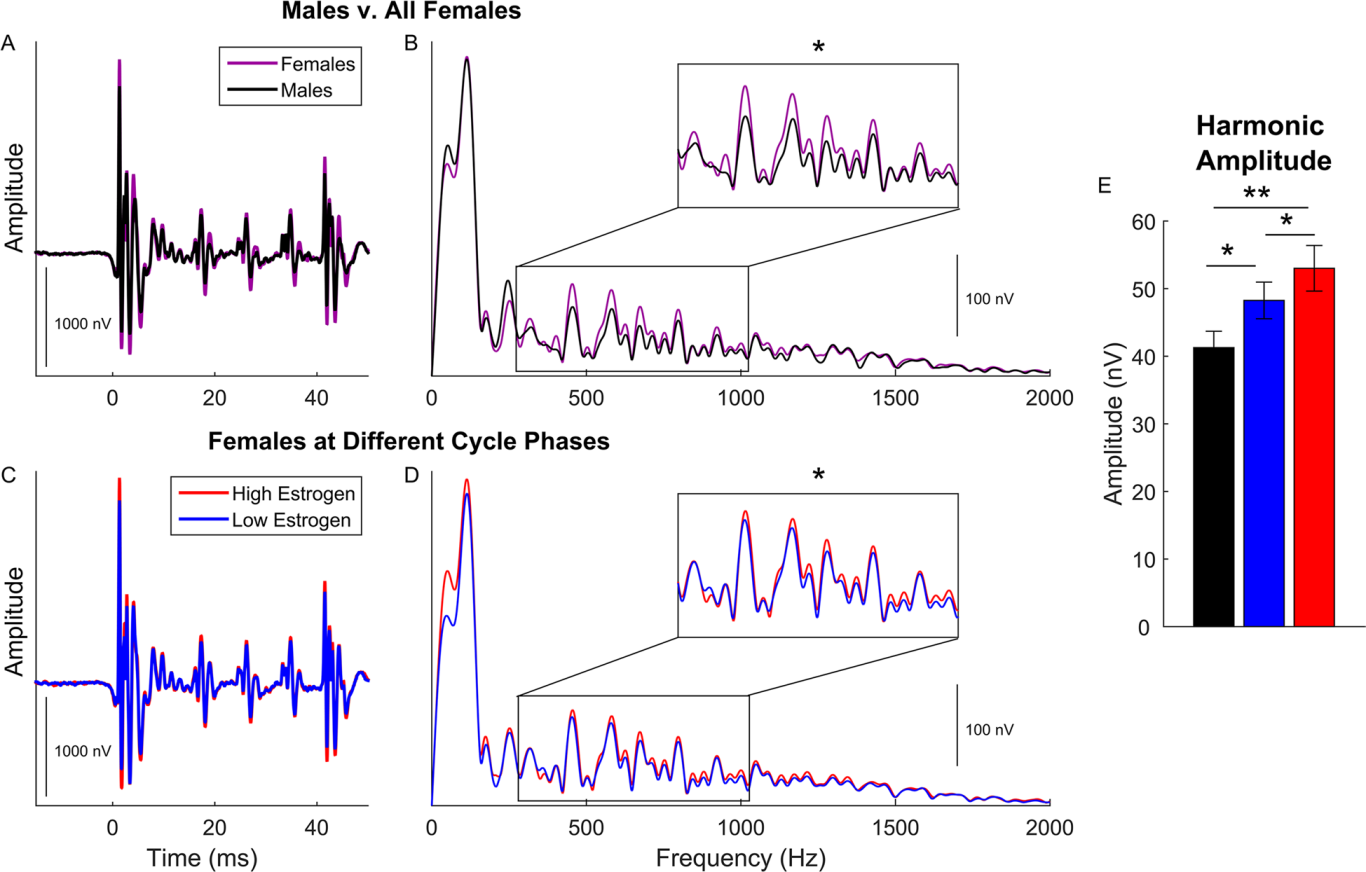
Time and frequency plots show harmonic encoding differences between male and female rats and variation in harmonic encoding across the female estrous cycle. Males (black) are plotted against all females (purple) in the time (**A**) and frequency (**B**) domains. The inset in B illustrates the frequency range of the harmonic analysis. This difference is specific to harmonic frequencies; no differences are seen in response to the fundamental frequency (75–160 Hz). In the bottom plots, within-subject comparisons of female responses are plotted in the time (**C**) and frequency (**D**) domains separately for recordings during proestrus/estrus (red), when estrogen levels were high, and during diestrus/metestrus (blue), when estrogen levels were low. The bar graph (**E**) plots the average ± 1 standard error harmonic amplitude for males (black), diestrus/metestrus females (blue), and proestrus/estrus females (red).

First, we determined whether female harmonic encoding enhancements previously reported in humans are evident in other mammals or specific to humans. FFRs of male rats were compared to female rats during two points in the females’ estrous cycle: once during metestrus/diestrus when circulating estradiol concentrations are low and once during proestrus/estrus when estradiol concentrations are high^10^. We found female rats had more robust encoding of the stimulus’ harmonic frequencies compared to male rats (Fig. 1A,B; ANOVA, F(2,21) = 5.141, *p* = 0.015, η_p_^2^ = 0.329; see Table 1 for means and standard deviations for all measures and groups) and that the difference between male and female rats was evident during both metestrus/diestrus (Fig. 1E; post-hoc t-test, t(14) = 2.224, *p* = 0.043, d = 1.11) and proestrus/estrus (Fig. 1E; post-hoc t-test, t(14) = 3.089, *p* = 0.008, d = 1.54) phases of the cycle.

**Table 1:** Means and standard deviations for each group on the 6 FFR measures.

Means and standard deviations						
Group	Harmonics (nV)	F0 (nV)	Neural noise (nV)	Response replicability (r)	Response magnitude (nV)	SNR
Females—high estrogen	53.01 (9.54)	284.76 (17.80)	138.8 (56.37)	.955 (.028)	497.46 (50.02)	3.93 (1.05)
Females—low estrogen	48.26 (7.67)	268.58 (51.57)	115.43 (28.33)	.959 (.031)	453.86 (81.90)	4.04 (0.72)
Males—Fall	40.14 (6.91)	281.36 (65.52)	153.79 (29.30)	.940 (.050)	482.67 (94.66)	3.29 (1.05)
Males—summer, test 1	40.82 (3.11)	274.09 (33.43)	138.60 (51.65)	.976 (.010)	492.29 (57.05)	3.86 (1.08)
Males—summer, test 2	39.53 (8.59)	287.29 (28.14)	129.02 (37.61)	.977 (.009)	500.21 (53.00)	4.06 (0.86)

Next, to determine whether harmonic encoding fluctuates with the estrous cycle, we compared female harmonic encoding within-subjects between proestrus/estrus and metestrus/diestrus. Females had greater encoding of harmonic frequencies when concentrations of estradiol are high than they did when estradiol concentrations are low (Fig. 1C,D; RMANOVA, F(1,7) = 9.916, *p* = 0.016, η_p_^2^ = 0.586).

**Figure 2:**
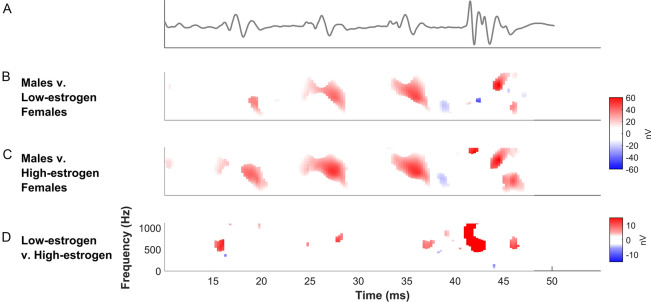
Spectrotemporal differences between males and females. For (**B**) and (**C**), red indicates greater energy at the given time and frequency for females and blue is greater energy for males. In (**D**), red corresponds to greater activity during estrus/proestrus and blue is for diestrus/metestrus. The top FFR waveform (**A**, gray) is zoomed in over the region of interest (16.5–43.5 ms). During this region, the larger peaks occurring every ~ 8.3 ms correspond to the periodicity of the fundamental frequency of the sound. These regions also correspond to where differences in harmonic encoding between males and metestrus/diestrus (**B**) and proestrus/estrus (**C**) exist. The temporal alignment of the fundamental periodicity and the harmonic enhancement suggest that the harmonic enhancement also provides females with greater tracking of the fundamental frequency. In contrast, the differences in harmonic encoding across the estrous cycle are most concentrated toward the end of the response (**D**), which suggests that sound boundaries are processed more robustly during estrus/proestrus.

To probe these differences further, we created difference spectrograms of the male and female FFRs to determine at what point in the stimulus these frequency-encoding differences were occurring. Comparing males to females, the differences occurred at the periodicity of the fundamental frequency (F0; Fig. 2A–C), suggesting that the harmonic enhancement also provides females with greater tracking of the fundamental frequency without having to enhance their response to the F0 specifically. Differences across the estrous cycle were mainly driven by enhanced harmonic encoding over the final ~ 10 ms of the response (Fig. 2D), which may suggest the difference is related to the encoding of boundaries of sounds (e.g., sound starts and stops) that are significant for auditory comprehension^11^.

We then investigated the specificity of this female frequency-encoding enhancement by comparing the amplitude of male and female responses to the fundamental frequency. No differences were seen in F0 amplitude (F(2,21) = 0.240, *p* = 0.789 , η_p_^2^ = 0.022), suggesting that the encoding differences were specific to harmonic frequencies.

Lastly, we determined that differences in harmonic encoding strength could not be explained by differences in neural noise (ANOVA, F(2,21) = 1.854, *p* = 0.181, η_p_^2^ = 0.150), overall response magnitude (ANOVA, F(2,21) = 0.649, *p* = 0.533, η_p_^2^ = 0.058), or replicability of the response across stimulus presentations (ANOVA, F(2,21) = 0.738, *p* = 0.490, η_p_^2^ = 0.066). FFRs from all rats were well above the noise floor, with signal-to-noise ratio (SNR) values ranging from 1.801 to 5.138 (M ± SD: 3.75 ± 0.975); and, SNR did not differ among the three groups (ANOVA, F(2,21) = 1.451, *p* = 0.257, η_p_^2^ = 0.121). Furthermore, male rat harmonic encoding from FFRs collected at two time points showed no differences (RMANOVA, (F(1,4) = 0.264, *p* = 0.634, η_p_^2^ = 0.062), establishing an expected test–retest reliability and suggesting that the female differences in harmonic encoding could not be explained by random variation across days. Female FFRs collected during proestrus/estrus and metestrus/diestrus did not differ on any other FFR measure (Table 2) and males tested at two time points showed no differences across recordings (Table 3).

**Table 2:** RMANOVA results for female FFRs on remaining measures.

Additional female comparisons across cycle				
FFR measure	F	df	p	η_p_^2^
F0	0.535	(1,7)	.488	.071
Neural noise	0.912	(1,7)	.371	.115
Response replicability	0.728	(1,7)	.422	.094
Response magnitude	2.726	(1,7)	.143	.280
SNR	0.035	(1,7)	.857	.005

**Table 3:** RMANOVA results for male FFRs on remaining measures.

Additional male test–retest comparisons				
FFR Measure	F	df	p	η_p_^2^
F0	0.988	(1,4)	.376	.198
Neural noise	0.080	(1,4)	.792	.020
Response replicability	4.353	(1,2)	.172	.685
Response magnitude	0.043	(1,4)	.846	.011
SNR	0.078	(1,4)	.794	.019

## Discussion

We conclude that females have more robust harmonic encoding than males; and, that the strength of harmonic encoding in females varies over the estrous cycle. The cyclical variation of harmonic encoding in females (Figs. 1C–E, 2D) suggests that changes in circulating estrogen over the course of the cycle likely underlie differences in harmonic encoding, both between males and females and within females. Indeed, previous work has found that systemic application of estrogen can lead to plasticity in auditory processing^7,9^ and coding fidelity for behaviorally-significant sounds^12^.

Although estrogen receptors ERα and ERβ are expressed throughout the adult mammalian auditory system^13^, the mechanism of action is likely through ERα, given that its expression in the auditory system is down-regulated by circulating estrogen levels^14^ and that increases in peripheral harmonic sensitivity in breeding midshipman females is mediated by ERα^9^. ERα is found in the nonlemniscal regions of the inferior colliculus^14^, the presumed predominant contributor to the midline scalp-recorded FFR^15–22^, suggesting that changes in harmonic encoding result from estrogenic effects on efferent signaling.

In the hypothalamus, estrogen increases dendritic spine formation through temporary downregulation of GABA-ergic inhibitory neurotransmission^23,24^ by decreasing the probability of GABA release^25^. As GABA application is known to decrease evoked responses to sound^26^, estrogen may be working similarly to block GABA-mediated inhibition in auditory evoked signaling. In support of the link between estrogen and GABA activity, gonadectomy in female rats results in greater GABA-mediated inhibition^27^. Moreover, estrogen’s effects on GABA signaling may work concertedly with estrogen-mediated changes in excitatory glutamatergic signaling found in the lemniscal, ascending pathway^28^, consistent with previous reports of estrogen’s effects on glutamatergic signaling in the nucleus accumbens^29^ and hippocampus^30^.

These findings support a role for hormones in regulating auditory differences between the sexes, consistent with previous work. In humans, hearing thresholds in women are generally better than in men but the extent of the difference differs with phase of menstrual cycle^31^. Spontaneous otoacoustic emissions, which arise from activity of hair cells in the cochlea, are more numerous in women than men, but this difference is absent during the first 2 weeks of the menstrual cycle^8^. And certain auditory perceptual skills, such as forward masking and temporal-interval discrimination, though equivalent in adults, differ between male and female adolescents, suggesting a pubertal hormone-mediated effect on auditory processing development^32^. While the hearing threshold and otoacoustic emission differences are peripheral in origin, here we show that central auditory processing can also be affected by estrogen signaling in rodents. These hormonal influences may account for previously reported differences in central processing of sound in male and female humans^8,33,34^.

## Methods

### Subjects

Subjects were age-matched, adult male (n = 13; 275–300 g on arrival) and female (n = 12; 175–200 g on arrival) Sprague–Dawley rats (Charles River Laboratories, Wilmington MA). All animals were individually housed in a colony room with a 12-h light/dark cycle. All procedures were approved and conducted in accordance with guidelines by the Institutional Animal Care and Use Committee at Rutgers, The State University of New Jersey and data reporting is in compliance with ARRIVE guidelines.

### Experimental design

#### Frequency-following response recording

The frequency following response (FFR) was elicited by a 40-ms synthesized complex sound “da” in anesthetized rats (ketamine-xylazine, K: 90 mg/kg, X:11 mg/kg, i.p.). The “da” consists of an initial noise burst and a formant transition between the consonant and the vowel. The F0 and the first three formants change linearly over the duration of the stimulus (F0: 103–125 Hz, F1: 220–720 Hz, F2: 1700–1240 Hz, F3: 2580–2500 Hz) while F4 and F5 remain constant at 3600 and 4500 Hz, respectively. Recordings were obtained from 8 male rats at a single time point. Recordings were obtained from 5 male rats and all female rats twice, with a minimum of 48 h and a maximum of 49 h between recordings. Female recordings were made at time points that corresponded to periods of high (proestrus/estrus) and low (metestrus/diestrus) levels of circulating estradiol^10^. The male rats with two recordings were tested during the summer, while the remaining animals were tested in late fall. Due to the potential for seasonal variation in auditory processing^6,9^, between-subject comparisons were only made between the males and females tested in the fall. The data from the summer-collected rats were used only to determine whether test–retest differences could explain differences across the estrous cycle in females.

Stimulus presentation and neural response recordings were carried out using BioSig RZ software (TDT Inc.) and MF1 multi-field magnetic speaker, RA4LI headstage, RA4PA preamp, RZ6 multiprocessor and PZ5 amplifier hardware (TDT Inc). Evoked potentials were recorded using a three-electrode configuration, with subdermal needle electrodes (1 kΩ) positioned at the midline along the head (active), immediately below the left pinna (reference), and the midline on the back of the neck (ground).

The “da” was presented at 70 dB SPL at rate of 10.9 Hz to the right ear through a speaker in an open field configuration. The “da” was presented at two different polarities that were 180 degrees out of phase relative to each other. Each polarity was presented in two blocks of 1500 sweeps, resulting in a total of 6000 trials (3000 per polarity). FFRs were epoched online over a 66 ms window that began 15.8 ms prior to stimulus onset. Neural activity was filtered online from 100 to 3000 Hz at 6 dB/oct.

#### Determination of estrous cycle phase

Immediately prior to each FFR recording, vaginal lavage samples were obtained from female subjects using standard procedures^10,35,36^. Briefly, 20 µl of sterile saline was pipetted over the vaginal opening to obtain a liquid sample from the area to observe cell types and distributions. Subsequently, the tip of the micropipette was shallowly inserted into the vagina and 20 µl of sterile saline was flushed into and back out of the vagina. The vaginal lavage sample was pipetted onto a glass microscope slide, allowed to dry, and visualized under a light microscope at 10 × magnification.

**Figure 3:**
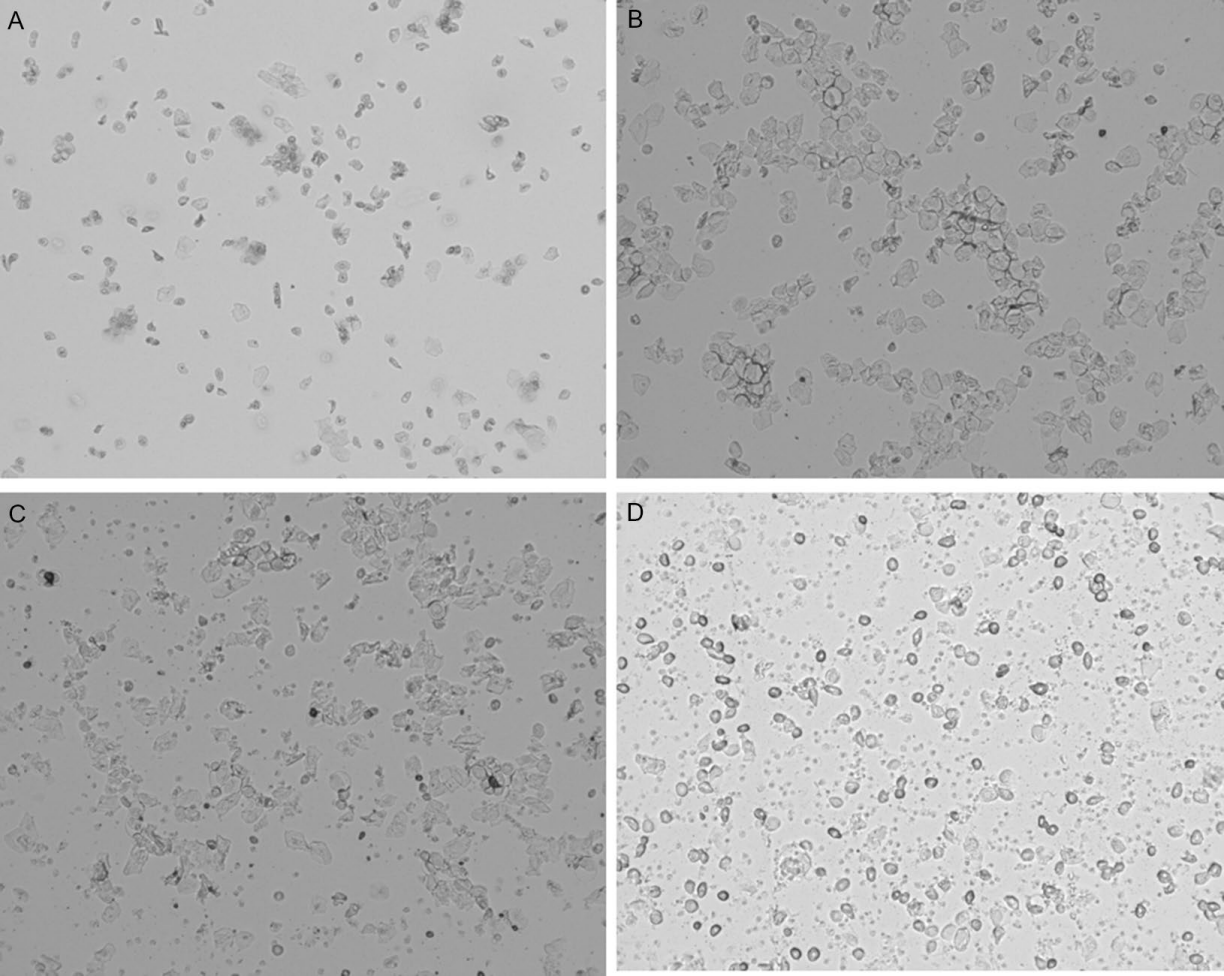
Representative vaginal lavage samples. Panels display samples rated as (**A**) proestrus, (**B**) estrus, (**C**) metestrus, and (**D**) diestrus.

The presence of certain cell types in the vaginal smear were used to determine cycle phase of each sample (Fig. 3). These determinations were made at the end of the study, after FFR data were collected, allowing the researchers to be blind to cycle stage during FFR collection. Proestrus was identified by the dominant presence of nucleated epithelial cells. Estrus was identified by the presence of primarily cornified cells. Metestrus was identified by the presence of roughly equal proportions of leukocytes, cornified cells, and nucleated epithelial cells. Diestrus was identified by an abundance of leukocytes. Up to 5 independent and trained raters evaluated each image to determine cycle phase by observed cell types and distributions in each sample. Only samples that were rated identically across 3 raters were included in further analyses. As such, electrophysiological data obtained on days without identifiable cycle phase and females without FFRs from cycle days corresponding to both low and high levels of circulating estrogen were excluded. These exclusions resulted in a final dataset of 8 female rats with recordings in both proestrus/estrus and metestrus/diestrus.

### Data processing

Data were processed using custom routines in Matlab. First, responses from the individual polarities were added together to accentuate the response to the stimulus envelope^37^, which shows the strongest sex difference in humans^1,5^. From these added responses, we extracted six measures: harmonic amplitude, fundamental-frequency (F0) amplitude, neural noise, broadband response magnitude, response replicability, and signal-to-noise ratio (SNR). Harmonic and F0 amplitude were analyzed using a fast Fourier analysis of the formant transition of ‘da’ (16.5–43.5 ms). The F0 amplitude was calculated by averaging activity from 75 to 160 Hz and the harmonics were averaged from 380 to 1020 Hz. These frequency regions were chosen to correspond with the fundamental frequency and first formant of the stimulus over this time period of the response, as well as higher harmonic frequencies between the first and second formant that have shown sex differences in humans^1,2^, and to capture the response to these frequencies across all recordings^38^. To calculate response replicability, 1500-sample averages from each polarity were added to create two 3000-sample responses. The two averages were compared via a Pearson product-moment correlation, where an r-value closer to 1 represents a more replicable response and an r-value nearer to zero reflects no replicability. To normalize these data, all data points were Fisher z-transformed prior to statistical analyses. Lastly, we calculated broadband response magnitude and neural noise as the root-mean-square amplitude over 16.5–43.5 ms of the response and the 15.8 ms pre-stimulus interval, respectively. SNR is the ratio of those two values.

### Statistical analyses

After ensuring that data met the correct assumptions for these analyses, to determine whether male and female rats differ in their auditory processing of complex sounds, an analysis of variance (ANOVA) was run on each measure comparing the fall-collected FFRs of males and the female FFRs collected during two phases of the estrous cycle (proestrus/estrus and metestrus/diestrus). Significant ANOVA’s were followed up with independent samples t-tests to compare the males to the females. A repeated-measures ANOVA (RMANOVA) was run to compare responses from females collected at the two points in the estrous cycle and to compare males tested twice during the summer.
