# Author Correction: Sex differences in auditory processing vary across estrous cycle

**DOI:** 10.1038/s41598-021-04143-5

**Published:** 2021-12-27

**Authors:** Jennifer Krizman, Elena K. Rotondo, Trent Nicol, Nina Kraus, Kasia M. Bieszczad

**Affiliations:** 1 Auditory Neuroscience Laboratory, Northwestern University, Evanston, IL 60208 USA; 2 Department of Communication Sciences and Disorders, Northwestern University, Evanston, IL 60208 USA; 3 Department of Neurobiology, Northwestern University, Evanston, IL 60208 USA; 4 Department of Otolaryngology, Northwestern University, Chicago, IL 60611 USA; 5 Department of Psychology—Behavioral and Systems Neuroscience, Rutgers, The State University of New Jersey, Piscataway, NJ 08854 USA

Correction to: *Scientific Reports* 10.1038/s41598-021-02272-5, published online 24 November 2021

The original version of this Article contained an error in the spelling of the author Kasia M. Bieszczad, which was incorrectly given as Kasia Bieszczad.

The original Article has been corrected.

